# A Desmoid Tumor Responding to Systemic Therapy With Tamoxifen and Sulindac

**DOI:** 10.7759/cureus.35723

**Published:** 2023-03-03

**Authors:** Hasan Choudhury, Oluseyi Abidoye

**Affiliations:** 1 Internal Medicine, Northeast Georgia Medical Center Gainesville, Gainesville, USA

**Keywords:** surgery, wnt, sulindac, tamoxifen therapy, desmoid-type fibromatosis

## Abstract

Desmoid tumors are locally aggressive benign tumors arising from connective tissue and are classified as soft tissue sarcomas that do not metastasize. The name is derived from the Greek word desmos that means tendon-like. These tumors are also known as aggressive fibromatosis and have an unpredictable natural history that varies depending on risk factors. They are treated as sarcomas because of their locally aggressive nature and a high local recurrence rate. The causes behind desmoid tumor development are enigmatic and their clinical course is unpredictable. Disease progression also varies widely depending on multiple syndromic risk factors. At this time, there is no scientific consensus over best treatment practices for this tumor type. Treatment can potentially be a combination of observation, systemic therapy, surgery or radiation therapy. Here, we have described a case of a female patient with a sporadic desmoid tumor that successfully responded to tamoxifen and sulindac.

## Introduction

Desmoid tumors are locally aggressive tumors classified as soft tissue sarcoma by the National Comprehensive Cancer Network (NCCN). Although these tumors are benign, they are classified as such because of their locally aggressive nature. These tumors are known to lack the potential for metastasis or dedifferentiation. Owing to their rarity, the desmoid tumor incidence rate is difficult to quantify. Some sources report an incidence rate of two to four cases per million individuals each year [1]. They have a high risk of recurrence, especially after surgical resection alone. Such recurrence leads to morbidity related to impingement of vital structures and organs. The causes behind their development are enigmatic and their clinical course is unpredictable. Disease progression depends on multiple syndromic risk factors. Major syndromic risk factors include a history of familial adenomatous polyposis (FAP) or Gardner syndrome [2]. This case report will focus on an instance of sporadic desmoid tumor after its initial post-operative recurrence that responded to tamoxifen and sulindac.

Desmoid tumors typically present as a painless mass noted in the extremity but they can arise in multiple body locations. For example, they can develop intra-abdominally and may therefore present with nausea, early satiety, and bowel obstruction with or without ischemia. Imaging with computed tomography (CT) or magnetic resonance imaging (MRI) helps define the tumor’s relationship with other anatomical structures. However, there are no radiographic features that can distinguish desmoids from malignant soft tissue tumors. A diagnosis is reached primarily with a histopathological examination, which typically shows pathognomonic monoclonal fibroblastic proliferation in a fibrous stroma [3]. Immunohistochemistry stains can also help with overall diagnosis. For example, high-level beta-catenin staining is a useful diagnostic marker [4]. The current literature indicates that two genes from the Wnt pathway, APC and CTNNB1, might be implicated in adult tumors [5,6]. Genomic analysis shows that mutations in this pathway are present in most patient samples. Other abnormal genetic associations include trisomy 8 and/or 20 [7].

Desmoid tumors behave unpredictably. They can grow overtime, but there have been periods of growth arrest/spontaneous regression in some studies [8]. However, there are no guidelines that establish “low-risk patients” who would benefit from observation. Disease recurrence depends on anatomic site, gender, size, and age. Cause-specific mortality has been reported to be 1% in patients with tumors in extra-abdominal sites [9,10].

Treatment for extra-abdominal desmoid tumors can include a combination of observation, surgery, radiation (alone or neoadjuvant) or systemic therapy (neoadjuvant or adjuvant) [11-15]. We report a case of a patient with sporadic desmoid fibromatosis who had recurrence following surgical resection starting at age 12. Given her recurrence, she was started on systemic therapy with tamoxifen and sulindac that has thus far prevented disease progression.

## Case presentation

The patient was a 30-year-old female with a past medical history significant for recurrent fibromas restricted to the left lower limb. The patient did not have any other medical problems or any family history of fibromatosis. Her first diagnosis with fibromas was at age 12 that was two years after the onset of menses. Overall, the patient had had approximately 13 recurrences at different locations in the left lower limb (including the thigh, popliteal area, and lower leg). It was noted that each time, the tumors seemed to surround a nerve bundle. The sciatic nerve and cutaneous nerves were involved on some occasions. Each time, the tumors were excised when they became large enough to cause compression symptoms. Symptoms included leg pain and cramps that were either spontaneous or triggered by compression upon sitting. She did not have any other associated systemic symptoms. Tissue exams were conducted each time. For the initial few masses, the biopsy results indicated neurofibromas because of the involvement of the neural sheath by the tumor. However, there were no other associated signs or symptoms to prove the condition to be neurofibromatosis. On recurrences that followed, it was diagnosed as a case of aggressive fibromatosis.

**Figure 1:**
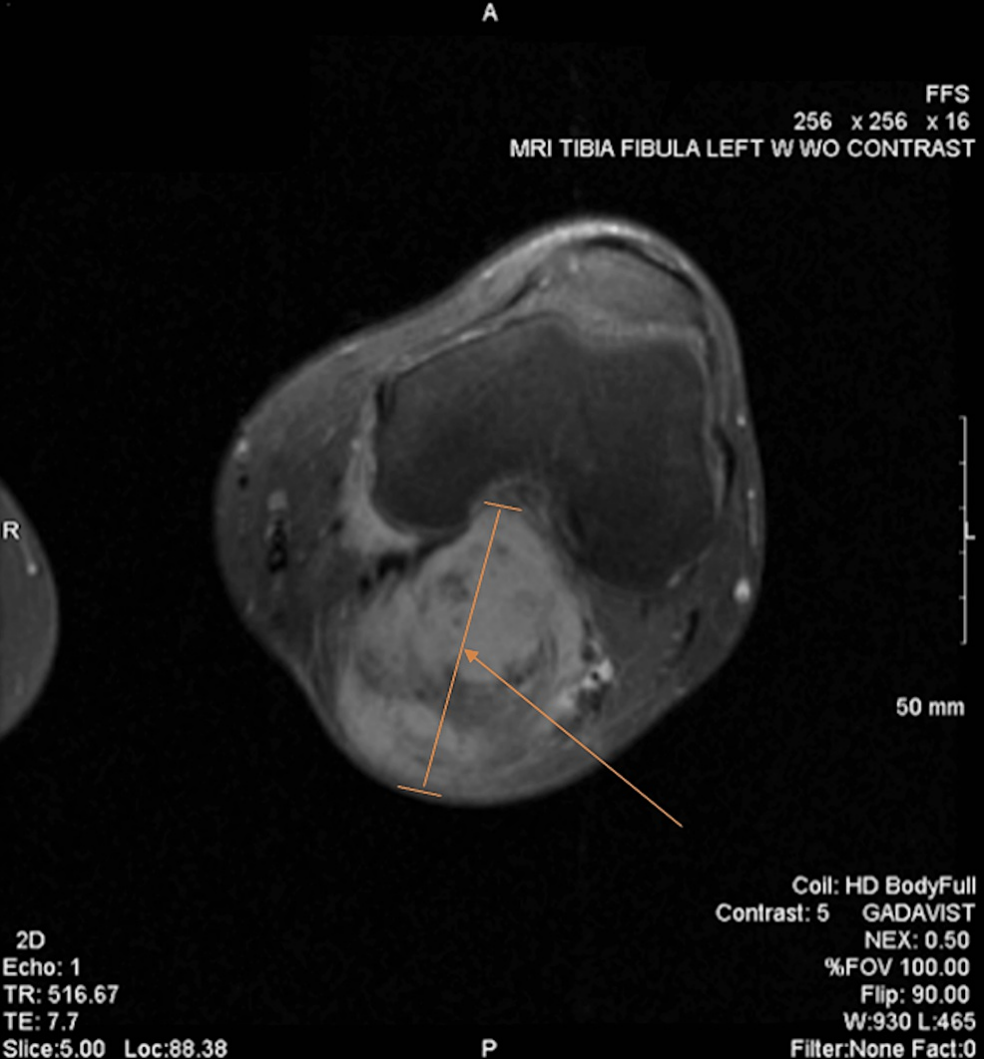
MRI (done in August 2020) demonstrating a transverse cut through the patella and distal end of the femur of the left leg. The arrow points to a line indicating the anterior-posterior length of the mass.

**Figure 2:**
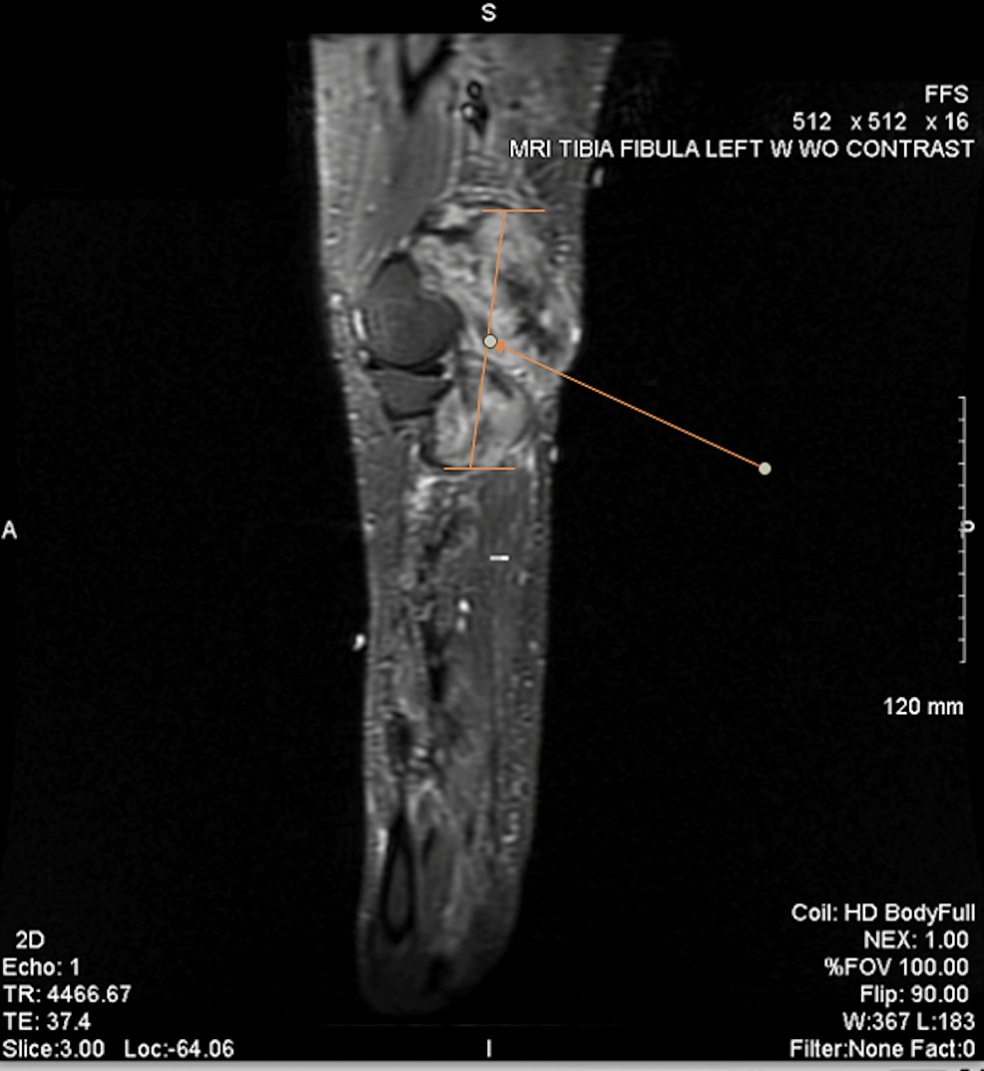
Sagittal view of the left leg demonstrating the mass posterior to the distal femur head A large heterogeneous mass can be seen in the popliteal fossa. The imaging study had shown the mass measuring 12 x 6.5 x 5.4 cm, extending inferiorly from the posterior thigh to the mid-calf with the epicenter of the mass within the popliteal fossa. The arrow points to a line that follows the cranio-caudal length of the mass.

**Figure 3:**
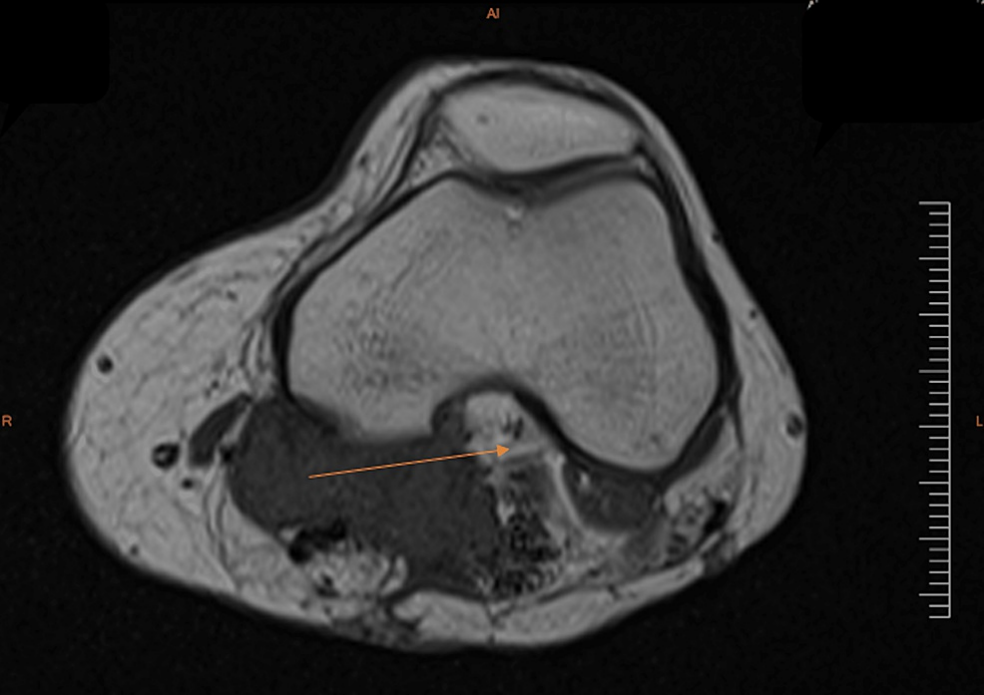
Post-operative MRI (done in December 2020) demonstrating a transverse cut through the patella and distal end of the femur of the left leg Post-surgical changes and a residual nodular mass measuring 4 x 2.5 cm were noted in the popliteal fossa. Avid enhancement was also seen along the popliteal neurovascular bundle. The arrow points to the post-operative mass that has been substantially reduced.

The most recent recurrence was in the year of 2019. At the time, the patient had a small mass in the left lower leg that was deemed too small to be operated on. This grew over a span of seven to eight months. An MRI scan obtained in August 2020 revealed the mass to be 12 x 6.5 x 5.4 cm (Figures 1, 2). The patient was evaluated by a plastic surgeon and underwent surgical excision of the mass in the popliteal region in August 2020. Given previous scar tissue in the region and extensive involvement of the neurovascular bundle, the mass could not be excised completely. The biopsy again confirmed desmoid fibromatosis. A post-operative MRI of the remaining mass tissue showed dimensions of 4 x 2.5 cm (Figure 3).

**Figure 4:**
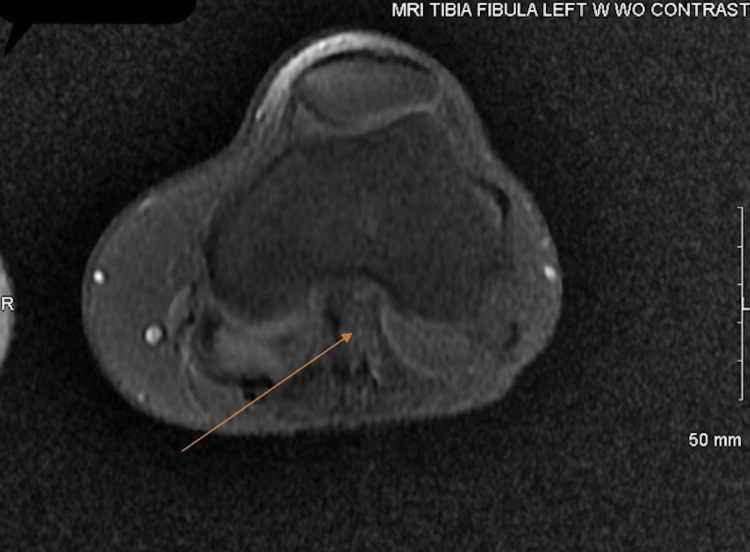
MRI (done in March 2022) after being on treatment for 15 months demonstrating a transverse cut through the patella and distal end of the femur of the left leg The mass (indicated by the arrow) at the popliteal fossa was slightly smaller (3.5 x 2.5 cm) when compared to the December 2020 scan and was again seen abutting the popliteal neurovascular bundle. No new abnormality or focal area of abnormal enhancement was seen.

The patient was then referred for follow-up with a medical oncologist and was started on tamoxifen 20 mg daily and sulindac 150 mg twice a day. She had surveillance MRI done every six months, and no changes in tissue dimensions were detected. After being on the medication for 15 months, MRI of the femur and tibia-fibula showed a 3.5 x 2.5 cm mass (Figure 4). She continued to have surveillance MRI scans at an interval of six to eight months and noted to have a stable exam each time for the last two years.

## Discussion

The case presented here is notable because it is about desmoid fibromatosis treated with systemic therapy that has thus far prevented tumor progression. This case can increase awareness regarding the importance of an interdisciplinary approach, given that these rare tumors can recur even after surgical resection regardless of positive or negative margins [16,17].

An important avenue for future studies includes focusing on the molecular pathways as a possible therapeutic target. Current treatments for desmoid tumors include tyrosine kinase inhibitors, hormonal therapy, anti-inflammatory agents, and cytotoxic chemotherapy [17]. A focus on the molecular pathways is interesting because this case shows that there are possibly hormonal factors that might influence the development of some types of desmoid tumors. Our case report suggests that this can at least be considered given that our patient's tumors began after the onset of menses and are now controlled with a selective estrogen receptor modulator (SERM). Therefore, it might be helpful to design a study to identify which patients will best respond to treatment with a SERM. For example, evaluating the response of a SERM in a patient population that had their first desmoid occurrence shortly after the onset of menses might have a higher response rate to a SERM versus an alternative systemic therapy with a different mechanism of action.

Additionally, the phase III placebo-controlled DeFi trial results in 2022 demonstrated that nirogacestat, a gamma-secretase inhibitor that targets the Notch pathway, led to improvements in progression-free survival (hazard ratio .20, 95% CI 0.15-0.55) [18]. This randomized controlled study showed a response rate of 41% in the treatment arm versus 8% in the placebo arm. This case highlights the heterogeneous nature of desmoid tumor pathophysiology. Similarly, it would be helpful to design studies that identify which patients will best respond to this treatment.

The scientific community could highly benefit from planning future trials comparing all the available therapeutics and that compare their relative efficacy. Such collaborative efforts would aid in the formulation of guidelines that could better direct clinical practices and treatment rubrics.

## Conclusions

This case highlights the importance of systemic therapy for desmoid tumors and increasing awareness of this rare disease so that medical treatments can be considered with or without surgery. This report is limited because it is a single case report. Nonetheless, given that there were no significant side effects, and with the overall positive response to therapy, future studies can evaluate the specific role of tamoxifen and sulindac at a molecular level. This could pave the way for additional targeted therapies. The morbidity associated with surgery for desmoid tumors suggests that such targeted medical therapies would be of particular benefit.
